# Nutrition Literacy of Portuguese Adults—A Pilot Study

**DOI:** 10.3390/ijerph18063177

**Published:** 2021-03-19

**Authors:** Mónica Monteiro, Tatiana Fontes, Cíntia Ferreira-Pêgo

**Affiliations:** 1 School of Sciences and Health Technologies, Universidade Lusófona de Humanidades e Tecnologias, Av. Campo Grande 376, 1749-024 Lisbon, Portugal; moniimonteiro12@gmail.com (M.M.); tatiana.fontes.97@gmail.com (T.F.); 2 CBIOS—Universidade Lusófona’s Research Center for Biosciences and Health Technologies, Av. Campo Grande 376, 1749-024 Lisbon, Portugal

**Keywords:** nutrition literacy, health literacy, Portuguese population, nutrition knowledge, health sciences

## Abstract

Nutrition is an essential factor in the prevention and treatment of some chronic diseases. For this reason, the population must know about nutrition, healthy food, and dietetics so that the promotion of healthier eating habits can lead to a consequent decrease in chronic disease incidence. That said, the present study aimed to assess nutrition literacy in the Portuguese population. Three hundred thirty participants aged between 18 and 65 years old were included in an observational, quantitative, and cross-sectional research. After the analysis, it was found that the vast majority of the study population (65.2%) had a good level of nutrition literacy. The participants having upper educational qualifications, following a specific diet, presenting an adequate BMI, having family members trained in the field of nutrition, and those who studied or worked in the field of health sciences reported a higher level of nutrition literacy. In conclusion, it seems to be essential to identify the population groups with the lowest nutrition knowledge so that it would be possible to apply personalized measures and to promote better literacy, reducing the prevalence and incidence of diseases and improving quality of life.

## 1. Introduction

Food plays a fundamental role not only in healthy growth and development but also in improving the quality of life, namely in the prevention of some chronic diseases [1]. It is estimated that more than half of premature deaths worldwide are due to chronic noncommunicable diseases, such as cancer, cardiovascular diseases, diabetes, or metabolic syndrome, which are largely influenced by the adoption of less healthy behaviors and lifestyles [2]. Chronic noncommunicable diseases are one of the most recurrent problems in public health in Portugal [3]. In 2015, approximately 29.7% of deaths in the Portuguese population were due to cardiovascular diseases [3], with stroke being more prevalent in Portugal than in all other European countries [4], while diabetes was common in approximately 10% of the Portuguese population, and high blood pressure was prevalent in approximately 36% [3]. In addition, chronic disease and a risk factor for developing others, obesity, together with overweight, affects more than half of the population [3]. Unhealthy eating habits are one of the main risk factors for these diseases in Portugal [4]. The increase in the prevalence of overweight is linked to overnutrition, where high energy consumption is associated with an insufficient intake of some nutrients [3]. Thus, nutrition is an essential factor for the prevention and treatment of chronic diseases, where a diet based on an ideal caloric intake, low intake of processed and red meats and also alcohol, and high consumption of fruits, vegetables, and whole grains is essential for the prevention of these pathologies [5]. In this way, the population must know about nutrition, healthy food and healthy habits, and dietetics so that the promotion of healthier eating habits may lead to a consequent decrease in chronic disease incidence [6]. The concept of health literacy, despite its constant evolution, is currently defined as the ability of individuals to obtain, process, understand, evaluate and use the concepts of health in order to make informed choices and thus reduce health risks and increase their quality of life [7,8]. The level of health literacy of the individual was already described to be mainly related to educational qualifications, socioeconomic level, ethnicity, living and working conditions, and also culture [9]. Individuals with lower health literacy levels demonstrated greater difficulties in understanding issues related to nutrition [10]. In this way, several authors consider nutrition literacy (NL) as a domain of health literacy [11]. That said, NL is defined as the individual’s ability to obtain, process, and understand nutritional information, as well as their ability to adopt appropriate, adequate, and healthy eating behaviors [12]. This encompasses three aspects: functional NL, which refers to basic literacy skills; interactive NL, which includes the cognitive and interpersonal communication skills needed to receive adequate nutritional information and apply it correctly; and, finally, critical NL, which implies having more complex cognitive skills, such as analyzing and interpreting scientific evidence [11]. Specifically, in Portugal, the National Program for the Promotion of Healthy Eating is one of the priority programs to be developed by governmental actions and designed by the ministry of health. This program mainly aims to improve the population’s nutritional status by encouraging the availability and consumption of foods that integrate a healthy eating regime [13]. However, to the best of our knowledge, there is no available evidence of published research that provides an understanding of food literacy, its determinants, and influential factors for the Portuguese population. For all these reasons, the main objective of the present analysis was to assess NL in a sample of Portuguese adults and to identify which sociodemographic variables are associated with this knowledge.

## 2. Materials and Methods

### 2.1. Population and Study Design

The present analysis consists of a cross-sectional observational pilot-study with the main aim of evaluating the NL of a sample of Portuguese adults. The total sample was composed of 338 participants living in Portugal and aged between 18 and 65 years. All participants, before data collection, agreed to participate in the study, giving their written and informed consent to participate. Before the filling of the questionnaire, the objective of the study and the variables to be evaluated were described, and the anonymity of the data was assured. This study was carried out following the ethical standards established in the 1964 Helsinki Declaration. The health and sciences technology school (ECTS) ethics committee approved the realization of the present study (EC.ECTS/P02.21) and declared the low risk for the population because the present analysis was an observational study without any type of intervention and all the data was collected online, having no presence of any time of human contact.

### 2.2. Nutrition Literacy Questionnaire

To assess the NL of the Portuguese population, the Nutritional Knowledge Questionnaire (NKQ) was used, translated from the one originally conceived and validated by Parmenter and Wardle in 1999 [14]. The questionnaire was applied through an online platform (Google^®^ Forms), between July 31st and October 8th, 2020, and its dissemination was carried out through different individual (researchers, collaborators and between others) and/or institutional (university, school of health, research center, dietitians associations, etc.) social networks (different newsletters, facebook^®^, instagram^®^, twitter^®^, whatsapp^®^, linkedin^®^, researchgate^®^, etc.) The questionnaire, consisting of 107 items, was divided into five parts. In the first part, questions related to the general characteristics of the participants were asked (age, gender, height, weight, area of residence, nationality, educational qualifications, professional situation, study or professional area, marital status, ethnicity, smoking habits, the existence of someone trained in nutrition in the family and its respective household, following a specific diet to improve health and, if so, which one, and finally reading habits of nutritional labels). Subsequently, the Body Mass Index (BMI) was calculated using the validated formula (weight/height^2^). The variables “Study Area” and “Work Area” were categorized into one, being designated by “Area of Performance”, divided into “health sciences” and “other areas”. The second part of the questionnaire made it possible to understand whether the participants had knowledge about the dietary recommendations given by the experts and consisted of 11 items, all of which were multiple-choice, with exception of one, and the score ranged between 0 and 11 points (giving 0 points if the condition was not meet, and giving 1 point for each condition meet). The third part of the survey made it possible to assess knowledge about the composition of certain foods and consisted of 69 items, most of which were multiple-choice and the others were “agree/disagree”, with the score ranging from 0 to 69 points (giving 0 points if the condition was not meet, and giving 1 point for each condition meet). The fourth part evaluated the participants’ ability to make healthy food choices, where 10 multiple-choice items were used, and it was possible to reach a maximum score of 10 points, with each question worth 1 point. Finally, the fifth and last section evaluated the ability to associate nutrition and health, where 5 questions were open-ended and the remaining 20 were multiple-choice, some questions worth 1 point and other 2 and 3 points, reaching 20 points. Therefore, the maximum questionnaire score was 110 points. For the classification of NL, the scores were categorized into four levels of literacy: between 0 to 30 points correspond to insufficient NL; between 31 to 60 points correspond to sufficient NL; between 61 to 90 points indicates good NL; and, finally, between 91 to 110 points equals very good NL.

### 2.3. Statistical Analysis

Statistical analysis was performed using the Statistical Package for Social Sciences (IBM SPSS) version 26.0 (SPPS Inc., Chicago, IL, USA). Data were presented as percentages (%) and absolute values (*n*) for dichotomous variables and mean and standard deviation (SD) for continuous variables. It was compared using the distribution of the selected characteristics between groups using Pearson χ^2^ tests for categorical variables and Student’s t-tests and analysis of variance (ANOVA), as appropriate, for continuous variables. All statistical tests were two-tailed and the significance level was set at *p* < 0.05.

## 3. Results

The sample under study was composed of 338 participants, 94 of whom were studying or working in the health sciences area, the remaining 236 in other different areas and eight individuals with missing values for this variable. According to Table 1, regarding the general characteristics of the study population, it was found that 85.2% of the participants were female and 14.8% male, with a mean age of 33.5 years, being those from health sciences significantly younger than those from other areas. No other sociodemographic statistical differences were found.

In Table 2, referring to the food-related characteristics of the study population, it was found that the vast majority of participants (88.5%) did not have family members trained in nutrition, existing statistically significant differences between the area of performance. It was also observed that 77.6% of the sample did not follow any specific diet, existing again statistically significant differences between the professional area.

The focus of this study was based on the assessment of NL, through a translated version of the validated NKQ questionnaire. That way, in Table 3 it can be observed that the vast majority of the study population (65.20%) had a good level of NL. It was also found, when analyzing the levels of literacy by professional area, that the participants in the health sciences area presented statistically better NL (72.3% of them presented very good and good level of NL), compared to other performance areas.

On the other hand, in Table 4, it was found that the participants who had family members trained in the nutrition sciences area presented the highest levels of NL (21.1%), with statistically significant differences compared to those who do not have nutrition experts as family members.

Through Table 5, it was possible to observe that the participants who had a higher BMI (≥25.0 kg/m^2^) had the highest values in the good level of NL. However, the sum of the very good and good levels demonstrated that the participants with a BMI between 18.5 and 24.9 kg/m^2^ had a better NL, with statistically significant differences between the BMI categories. It is also important to note that those participants with BMI below 18.5 Kg/m^2^ presented more individuals in the insufficient literacy category.

In Table 6, it can be observed that the total score was statistically different between the several areas of living, being the Azores archipelago the area with the highest score in the NL questionnaire, while the Lisbon Metropolitan area presented the lowest score.

In Table 7, it was observed that the university graduated participants obtaining a higher total score in the questionnaire (71.3%), with statistically significant differences compared to the rest of the educational qualifications.

Finally, in Table 8, referring to the level of NL according to whether or not to follow specific diets there were no statistically significant relations. However, the NL score was slightly higher in those individuals following a specific diet.

## 4. Discussion

To the best of our knowledge, the present study was the first one to describe that half of the Portuguese adults evaluated presented a good level of NL, and it was also the first to describe that this literacy was conditioned by having upper educational qualifications, following a specific dietetic pattern, presenting an adequate BMI, having family members trained in the field of nutrition, and by the fact of studying or working in health sciences area. Similar results were already described by other researchers, concluding that participants from less favored backgrounds, with possible economic, social, language, and cultural barriers, tend to have lower educational qualifications and, consequently, lower NL [15]. Meeting the results obtained in the present analysis, another Portuguese study found that approximately half of its sample demonstrated a good and higher degree of NL [16]. This can be explained by the fact that, nowadays, people, especially those of younger ages, find it easier to access the internet and, consequently, find it easier to seek information about nutrition and healthy habits [17]. Furthermore, a study in Iran found that nutrition knowledge was lower in participants from areas other than health sciences, such as, for example, participants in the area of business administration [18]. Geaney and coworkers found that individuals from the health sciences field, especially from dietetics and nutrition, had a higher score of nutrition knowledge [19]. Drichoutis and colleagues also corroborate this information, reporting that individuals who do not obtain nutritional formation through specialties, such as nutritionists and dietitians, have less nutritional knowledge [20]. This is because, in courses and jobs related to health sciences, mainly nutrition and dietetics, topics like nutrition, healthy eating, and correct eating habits are regularly addressed, as well as the people who are accompanied by these professionals [17]. Contrary to what was obtained in the present analysis, several studies did not observe statistically significant differences between the level of NL and BMI, suggesting that there may exist other factors than nutrition knowledge influencing BMI [21,22]. However, this is an important topic and should be explored in future research lines. Additionally, in the present study, participants from the Azores archipelago showed a higher total score in NL, which contradicts the latest data from the National Statistics Institute which reveal that, in Portugal, it is in the Autonomous Region of the Azores that they present the lowest educational levels [23], and several studies point to the relationship between a higher level of NL and the education degree [19,24]. More studies are needed to better understand and explain this relation, but probably these results should be attributable to the differences between the sample distribution between these categories of analysis (220 in Lisbon area vs. 5 in Azores archipelago), and for this reason, the results could be unreliable. Moreover, Drichoutis and coworkers suggested that there is a relationship between high levels of NL and the follow-up of specific diets or dietary patterns because people who seek to adopt this type of diet follow advice from health professionals who are experts in the field, namely nutritionists and dietitians [20]. Regarding the influence of nutrition literacy on food choices, results from a meta-analysis of the experimental studies suggested that the addition of contextual or interpretive nutrition information on menus, increasing literacy in nutrition and dietetics area, appeared to assist consumers in the selection and consumption of fewer calories [25]. Another study, carried out in adults, showed that individuals with a low level of nutritional literacy had a lower predisposition to consume the recommended daily amount of fruits and vegetables, as well as to read the food labels properly [10]. On the other hand, Teixeira et al. found that the realization of a theoretical and practical intervention on nutritional literacy, in two months, resulted in a change in eating behavior and in the reduction of body weight in the group of individuals under study [26]. Although the present study still presents a considerable sample (but not representative), its heterogenicity in number and gender distribution is the main limitation and does not allow results extrapolation and generalization. Another limitation refers to the questionnaire and the fact that it is quite extensive, which may have led participants to answer less carefully in the last questions. In addition, the tool was not validated in the Portuguese language, nor Portuguese culture and cuisine, which could affect the results obtained because the reliability of this tool was not rated. The cross-sectional design is also a limitation because it does not allow to establish a causal relation. It should also take into account a possible limitation on the fact that individuals who answered this questionnaire were initially individuals interested in the nutrition field and a special interest in this area of study. Nevertheless, some strengths should also be noted, like, for example, the use of a validated and published questionnaire. Finally, further studies should be carried out to clarify this relationship between levels of NL and the several general and sociodemographic characteristics of the population.

## 5. Conclusions

The vast majority of the Portuguese adults included in this analyzed sample had satisfactory knowledge about the nutrition area. However, those who studied or worked in the field of health sciences, those had family members with a background in nutrition, those who had an adequate BMI, those who presented higher academic degree and those following a specific dietary pattern were the participants who demonstrated having a higher level of NL. In this way, it is extremely important to identify the population groups where the nutrition knowledge is lower and to pay more attention to those groups of individuals with less knowledge about this area identified in the present study, to create and implement better and more adequate and specific nutrition education programs and actions in a way to improve eating behaviors and food choices. Nutrition literacy of the Portuguese adults, and population in general, should be more actively taken into consideration by policymakers because it is a way to improve the population’s lifestyle and to reduce the incidence of several chronic diseases and improve present and future health.

## Figures and Tables

**Table 1:** General characteristics of the studied population categorized by professional area.

	Total Population (*n* = 330)	Health Sciences (*n* = 94)	Other Areas (*n* = 236)	*p*-Value *
**Gender, %(*n*)**				
Male	14.8 (49)	9.6 (9)	16.9 (40)	0.089
Female	85.2 (281)	90.4 (85)	83.1 (196)	
Age, years	33.5 (11.8)	30.1 (9.7)	34.9 (12.4)	0.001
Height, m	1.7 (0.1)	1.7 (0.1)	1.7 (0.1)	0.782
Weight, kg	67.4 (13.6)	65.3 (13.5)	68.2 (13.5)	0.077
BMI, kg/m^2^	24.7 (5.1)	23.9 (4.9)	25.1 (5.1)	0.062
**BMI classification, %(*n*)**				
<18.5 kg/m^2^	7.3 (24)	9.6 (9)	6.4 (15)	0.450
18.5–24.9 kg/m^2^	52.4 (173)	54.3 (51)	51.7 (122)	
≥25 kg/m^2^	40.3 (133)	36.2 (34)	41.9 (99)	
**Smoking Habits, %(*n*)**				
Smoker	13.0 (43)	10.6 (10)	14.0 (33)	0.088
Ex-smoker	13.0 (43)	7.4 (7)	15.3 (36)	
Non Smoking	73.9 (244)	81.9 (77)	70.8 (167)	
**Marital status, %(*n*)**				
Single	53.0 (175)	64.9 (61)	48.3 (114)	0.052
Married	41.8 (138)	31.9 (30)	45.8 (108)	
Divorced	4.8 (16)	3.2 (3)	5.5 (13)	
Widower	0.3 (1)	0.0 (0)	0.4 (1)	
**Area of residence, %(*n*)**				0.075
North	14.8 (49)	23.4 (22)	11.4 (27)	
Center	9.7 (32)	9.6 (9)	9.7 (23)	
Lisbon Metropolitan Area	64.8 (214)	57.4 (54)	67.8 (160)	
Alentejo	2.1 (7)	1.1 (1)	2.5 (6)	
South	2.1 (7)	2.1 (2)	2.1 (5)	
Madeira archipelago	4.8 (16)	3.2 (3)	5.5 (13)	
Azores archipelago	1.5 (5)	3.2 (3)	0.8 (2)	
**Academic degree, %(*n*)**				
≤Secondary education	39.1 (129)	29.8 (28)	42.8 (101)	0.068
University graduation	41.8 (138)	45.7 (43)	40.3 (95)	
Postgraduate studies	19.1 (63)	24.5 (23)	16.9 (40)	
**Professional situation, %(*n*)**				
Employee	55.2 (182)	46.8 (44)	58.5 (138)	0.065
Student	28.8 (95)	39.4 (37)	24.6 (58)	
Working-student	5.8 (19)	5.3 (5)	5.9 (14)	
Unemployed	10.3 (34)	8.5 (8)	11.0 (26)	

Data expressed as a percentage (*n*) or mean (SD) for categorical or continuous variables, respectively. * *p*-Value, for comparisons between groups, was tested by the *t*-Student test or Pearson’s χ^2^ test, as appropriate. Abbreviations: BMI, Body Mass Index.

**Table 2:** Food-related characteristics according to the professional area.

	Total Population (*n* = 330)	Health Sciences (*n* = 94)	Other Areas (*n* = 236)	*p*-Value *
**Family member trained in nutrition, %(*n*)**	11.5 (38)	19.1 (18)	8.5 (20)	0.006
**Following some specific diet, %(*n*)**	22.4 (74)	13.8 (13)	25.8 (61)	0.018
**Habit of reading food labels, %(*n*)**				
Yes	12.7 (42)	8.5 (8)	14.4 (34)	0.464
Sometimes	80.6 (266)	85.1 (80)	78.8 (186)	
No	6.4 (21)	6.4 (6)	6.4 (15)	
I don’t know what a food label is	0.3 (1)	0.0 (0)	0.4 (1)	

Data expressed as a percentage (*n*). * *p*-Value, for comparisons between groups, was tested by Pearson’s χ^2^ test.

**Table 3:** The literacy level of the population studied according to the professional area.

	Total Population (*n* = 330)	Health Sciences (*n* = 94)	Other Areas (*n* = 236)	*p*-Value *
**Total Score**	68.7 (14.8)	71.4 (16.4)	67.6 (14.0)	0.031
**Literacy level** **, %(*n*)**				
Very good	6.1 (20)	13.8 (13)	3.0 (7)	0.003
Good	65.2 (215)	58.5 (55)	67.8 (160)	
Sufficient	27.3 (90)	26.6 (25)	27.5 (65)	
Insufficient	1.5 (5)	1.1 (1)	1.7 (4)	

Data expressed as a percentage (*n*) or mean (SD) for categorical or continuous variables, respectively. * *p*-value, for comparisons between groups, was tested using the *t*-Student test or Pearson’s χ^2^ test, as appropriate.

**Table 4:** The literacy level of the population studied according to whether they have family members in the nutrition area.

	Total Population (*n* = 338)	Yes (*n* = 38)	No (*n* = 300)	*p*-Value *
**Total Score**	68.6 (14.71)	76.7 (2,35)	67.6 (14.44)	<0.001
**Literacy level, %(*n*)**				
Very good	5.9 (20)	21.1 (8)	4.0 (12)	<0.001
Good	65.7 (222)	65.8 (25)	65.7 (197)	
Sufficient	26.9 (91)	13.2 (5)	28.7 (86)	
Insufficient	1.5 (5)	0.0 (0)	1.7 (5)	

Data expressed as a percentage (*n*) or mean (SD) for categorical or continuous variables, respectively. * *p*-value, for comparisons between groups, was tested using the *t*-Student test or Pearson’s χ^2^ test, as appropriate.

**Table 5:** The literacy level of the population studied according to BMI categories.

	Total Population (*n* = 338)	<18.5 kg/m^2^ (*n* = 24)	18.5–24.9 kg/m^2^ (*n* = 180)	≥25.0 kg/m^2^ (*n* = 134)	*p*-Value *
**Total Score**	68.6 (14.7)	68.4 (22.0)	70.1 (14.8)	66.7 (12.7)	0.124
**Literacy level, %(*n*)**					
Very good	5.9 (20)	25.0 (6)	6.7 (12)	1.5 (2)	< 0.001
Good	65.7 (222)	33.3 (8)	67.2 (121)	69.4 (93)	
Sufficient	26.9 (91)	37.5 (9)	24.4 (44)	28.4 (38)	
Insufficient	1.5 (5)	4.2 (1)	1.7 (3)	0.7 (1)	

Data expressed as a percentage (*n*) or mean (SD) for categorical or continuous variables, respectively. * *p*-value, for comparisons between groups, was tested using the *t*-Student test or Pearson’s χ^2^ test, as appropriate.

**Table 6:** The literacy level of the population studied according to a place of living.

	Total Population (*n* = 338)	North (*n* = 49)	Center (*n* = 33)	Lisbon (*n* = 220)	Alentejo (*n* = 7)	South (*n* = 8)	Madeira (*n* = 16)	Azores (*n* = 5)	*p*-Value *
**Total Score**	68.6 (14.7)	72.9 (14.4)	72.3 (14.9)	66.65 (14.4)	71.6 (11.7)	73.0 (13.2)	67.1 (15.3)	85.0 (15.3)	0.008
**Literacy level** **, %(*n*)**									
Very good	5.9 (20)	12.2 (6)	9.1 (3)	3.2 (7)	14.3 (1)	12.5 (1)	0.0 (0)	40.0 (2)	0.078
Good	65.7 (222)	69.4 (34)	63.6 (21)	65.0 (143)	71.4 (5)	75.0 (6)	68.8 (11)	40.0 (2)	
Sufficient	26.9 (91)	18.4 (9)	27.3 (9)	30.0 (66)	14.3 (1)	12.5 (1)	25.0 (4)	20.0 (1)	
Insufficient	1.5 (5)	0.0 (0)	0.0 (0)	1.8 (4)	0.0 (0)	0.0 (0)	6.3 (1)	0.0 (0)	

Data expressed as a percentage (*n*) or mean (SD) for categorical or continuous variables, respectively. * *p*-Value, for comparisons between groups, was tested using the *t*-Student test or Pearson’s χ^2^ test, as appropriate.

**Table 7:** The literacy level of the population studied according to educational qualifications.

	Total Population (*n* = 338)	≤Secondary Education (*n* = 131)	University Graduation (*n* = 143)	>Licentiate Degree (*n* = 131)	*p*-Value *
**Total Score**	68.6 (14.71)	65.5 (15.10) ^a^	71.3 (13.62) ^b^	69.2 (15.26)	0.005
**Literacy level** **, %(*n*)**					
Very good	5.9 (20)	3.8 (5)	7.0 (10)	7.8 (5)	0.138
Good	65.7 (222)	61.1 (80)	67.8 (97)	70.3 (45)	
Sufficient	26.9 (91)	32.8 (43)	25.2 (36)	18.8 (12)	
Insufficient	1.5 (5)	2.3 (3)	0.0 (0)	3.1 (2)	

Data expressed as a percentage (*n*) or mean (SD) for categorical or continuous variables, respectively. * *p*-Value, for comparisons between groups, was tested using the *t*-Student test or Pearson’s χ^2^ test, as appropriate. Bonferroni post-hoc differences between ^a^ and ^b^.

**Table 8:** The literacy level of the population studied according to following or not specific dietary patterns.

	Total Population (*n* = 338)	Following Diets (*n* = 75)	Not Following Diets (*n* = 263)	*p*-Value *
**Total Score**	68.6 (14.71)	69.0 (14.79)	68.5 (14.71)	0.809
**Literacy level** **, %(*n*)**				
Very good	5.9 (20)	4.0 (3)	6.5 (17)	0.733
Good	65.7 (222)	70.7 (53)	64.3 (169)	
Sufficient	26.9 (91)	24.0 (18)	27.8 (73)	
Insufficient	1.5 (5)	1.3 (1)	1.5 (5)	

Data expressed as a percentage (*n*) or mean (SD) for categorical or continuous variables, respectively. * *p*-Value, for comparisons between groups, was tested using the *t*-Student test or Pearson’s χ^2^ test, as appropriate.

## Data Availability

The study did not report any data.
